# Les garrots de prélèvement, un drame chez le nourrisson: à propos de 3 cas

**DOI:** 10.11604/pamj.2016.23.68.8224

**Published:** 2016-03-07

**Authors:** Edgar Ouangré, Moussa Bazongo, Isso Ouédraogo, Maurice Zida, Daouda Ouedraogo, Adama Sanou, Gilbert Patindé Bonkoungou, Rodrigue Namékinsba Doamba, Nayi Zongo, Si Simon Traore

**Affiliations:** 1 Service de Chirurgie Générale et Digestive du CHU-Yalgado Ouedraogo, 03 BP 7022 Ouagadougou, Burkina Faso; 2 Service de Chirurgie CHU Pédiatrique Charles de Gaulle, 01 BP 1198 Ouagadougou, Burkina Faso; 3 Service de Chirurgie Générale et Digestive du CHU de Tengandgo, 11 BP 104 Ouagadougou, Burkina Faso

**Keywords:** Garrot, gangrène ischémique, membre, amputation, Tourniquet, ischemic gangrene, limb, amputation

## Abstract

Le délai pour la levée d'un garrot sur un membre est limité, tout retard, surtout après la 3^ème^ heure expose à un risque d'amputation de celui-ci. Notre objectif a été de rapporter trois cas de gangrène ischémique de membre par oubli d'un garrot après un prélèvement sanguin, afin d'interpeler le personnel soignant sur ses dangers. Il s'est agi de trois nourrissons dont deux de 3 mois et un de 5 mois, reçus aux urgences viscérales du CHU-Yalgado Ouédraogo pour tuméfaction du membre thoracique gauche. Dans leurs antécédents, on a noté une pose de garrot pour prélèvement de sang qui a été oublié pendant 24 heures dans deux cas et 48 heures dans un cas. L'examen avait retrouvé un œdème diffus associé à un sphacèle du membre supérieur remontant jusqu'au 1/3 moyen du bras; une abolition des pouls ulnaire et radial ainsi que de la sensibilité de la main dans 2 cas. Dans un cas les signes étaient atténués. Le diagnostic de gangrène ischémique de membre a été retenu dans tous les cas. La biologie réalisée était normale. En urgence, il a été réalisé une amputation trans-humérale dans 2 cas et un débridement associé à une amputation de quatre doigts dans un cas. L’évolution a été simple dans tous les cas. La gangrène sèche iatrogène de membre par garrot en milieu hospitalier ne devrait pas se concevoir. Cela passe par la rigueur dans l'administration des soins et une surveillance régulière et attentive des patients.

## Abstract (en)

The time limit for the removal of a tourniquet is short; any delay in tourniquet deflation, especially if it exceeds the 3 hour limit, exposes to amputation hazards. Our objective was to report three cases of ischemic limb gangrene, caused by having forgotten to take a tourniquet off after a blood sampling, to inform healthcare professionals about the risk associated with that negligence. We encountered 3 cases of infants (2 three-month-old infants and 1 five-month-old infant), hospitalized in intensive care unit of Yalgado Ouédraogo University Hospital for upper-left limb swelling. Their medical history shows that there was a delay in tourniquet deflation after a blood sampling of 24 hours in two cases and of 48 hours in one case. Physical examination revealed a diffuse edema associated with upper limb gangrene spread to the mid-third of the upper arm, abolition of the ulnar and radial pulse as well as loss of sensation in the hand in 2 cases. In one case clinical signs were attenuated. The diagnosis of ischemic limb gangrene was confirmed in all cases. Laboratory examinations were normal. Two cases needed urgent trans-humeral amputation and one case needed debridement plus amputation of four fingers. The evolution was simple in all cases. Iatrogenic dry gangrene caused by a delay in tourniquet removal should never happen at hospitals. This can be guaranteed only by tightening up health management and by performing regular and accurate patient monitoring.

## Introduction

Le garrot est un lien serré autour d'un membre et dont le but est d'interrompre ou de ralentir la circulation sanguine [1]. Il est principalement connu comme un instrument utilisé en chirurgie pour limiter le saignement per opératoire, en médecine d'urgence comme technique de sauvetage en cas de saignement non accessible à la compression ou à une autre technique d'hémostase, et en cas d'afflux de victimes [2]. En outre il permet de faciliter le prélèvement de sang et la prise de voie veineuse au niveau des membres. Le délai pour sa levée étant limité, tout retard, surtout après la 3^ème^ heure expose à un risque d'amputation du membre [3]. Dans les pays en développement, on observe en milieu hospitalier avec récurrence les complications liées au garrot [4]. La faute médicale, la négligence dans la pratique de la médecine et l'ignorance des parents peuvent constituer une circonstance favorisante son oubli. Le but de notre étude a été d'apporter les circonstances de survenues de gangrène ischémique du membre supérieur chez trois nourrissons, afin d'interpeller le personnel soignant sur les dangers de l'oubli du garrot.

## Patient et observation

### Observation 1

**Figure 1:**
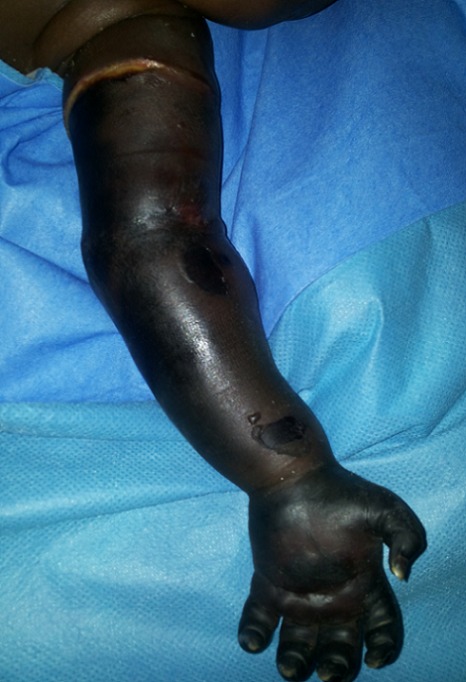
Gangrène ischémique du membre thoracique gauche avec un aspect carbonisé de la peau associé à des phlyctènes

NK nourrisson de 5 mois, de sexe masculin a été reçu le 22/ 01/ 2014 aux urgences viscérales du CHU- Yalgado Ouédraogo pour une modification de la coloration cutanée du membre thoracique gauche. Le patient avait consulté dans un centre médical en périphérie pour des douleurs abdominales. Dans le cadre de sa prise en charge d'une malnutrition aiguë, il a bénéficié d'une prise d'une voie veineuse et de prélèvements sanguins par un infirmier durant lesquels le garrot du prélèvement a été oublié pendant 48 heures. Devant la modification de la coloration cutanée des doigts, les parents ont alerté le personnel paramédical qui a procédé à l'ablation du garrot et à l’évacuation du nourrisson au CHU-Yalgado Ouédraogo. La mère du nourrisson était au courant de la présence du garrot après le prélèvement sanguin mais ne connaissait pas l'utilité de celui-ci. L'examen a noté, un œdème, des phlyctènes et des placards de sphacèle allant jusqu’à la racine du membre thoracique gauche avec une limite annulaire nette (Figure 1), une absence de pouls et une anesthésie du membre thoracique gauche. Ailleurs on a noté une fontanelle antérieure déprimée, des plis de déshydratation et de dénutrition. Le membre thoracique controlatéral était d'aspect normal. Le diagnostic de gangrène ischémique du membre supérieur gauche par oubli de garrot sur terrain de malnutrition aiguë modérée a été retenu. Le bilan biologique pré opératoire était normal. Il a été réalisé une amputation trans-humérale gauche au 1/3 supérieur. Les suites ont été simples avec une récupération nutritionnelle et une cicatrisation du moignon d'amputation.

### Observation 2

**Figure 2:**
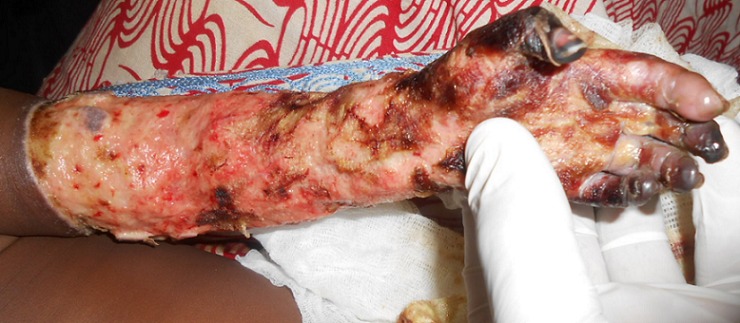
Gangrène ischémique des doigts avec un aspect ulcéro-nécrotique de la peau du membre thoracique gauche

K.A.A nourrisson de 3 mois, de sexe masculin a été reçu le 22/02/2014 aux urgences viscérales du CHU- Yalgado Ouédraogo pour un œdème associé à une cyanose du membre thoracique gauche. Il avait été hospitalisé dans un Centre de Santé et de Promotion Sociale (CSPS) pour une hyperthermie. Dans le cadre du bilan biologique, il a bénéficié d'un prélèvement sanguin par un infirmier après pose d'un garrot au bras gauche qui a été oublié. Vingt quatre heures après le prélèvement apparait une modification de la coloration cutané et un œdème. Les agents de santé ont été alertés par la mère, mais l'infirmier aurait ignoré la présence du garrot. Le nourrisson a été référé au Centre Médical avec Antenne chirurgicale (CMA) de la dite ville où le garrot a été découvert. L'examen du membre thoracique gauche avait noté, des lésions d'ulcéro-nécrotiques superficielles allant de la main jusqu’à la racine du membre avec une limite annulaire nette et une nécrose ischémique des doigts I, III, IV, V (Figure 2). Les pouls, la motricité et la sensibilité étaient conservés sauf au niveau des I, III, IV, et V doigts. Le diagnostic de gangrène ischémique du membre thoracique gauche a été retenu. Le bilan biologique préopératoire était normal. Il a été réalisé un débridement associé à une désarticulation métacarpo-phalangienne des I, III, IV et V doigts gauche. Les suites ont été simples.

### Observation 3

**Figure 3:**
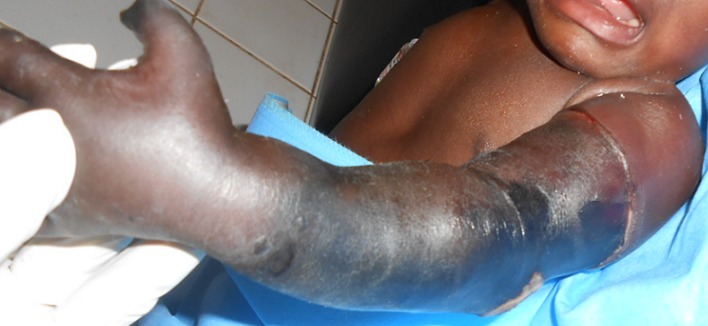
Gangrène ischémique avec œdème du membre thoracique gauche

M.H nourrisson de 3 mois, de sexe masculin a été reçu le 26/03/2014 aux urgences viscérales du CHU- Yalgado Ouédraogo pour un œdème associé à une modification de la coloration cutanée du membre thoracique gauche. Le patient avait consulté dans un Centre de Santé et de Promotion Sociale (CSPS) en périphérie pour une fièvre. Il a bénéficié de la prise d'une voie veineuse par un infirmier après pose d'un garrot au bras gauche qui a été oublié. Vingt quatre heures après, alerté par les parents, le garrot a été ôté puis le patient a été conduit au Centre médical avec antenne chirurgical d'où il a été é référé au CHU Yalgado Ouédraogo. L'examen a noté, un œdème, des phlyctènes et des placards de sphacèle allant jusqu’à la racine du membre avec une limite annulaire nette, une absence de pouls et une anesthésie du membre supérieur gauche (Figure 3). Le diagnostic de gangrène ischémique du membre thoracique gauche a été retenu. Le bilan biologique préopératoire était normal. Il a été réalisé une amputation trans humérale gauche. Les suites ont été simples.

## Discussion

Le garrot a été l’étiologie de la gangrène ischémique du membre thoracique gauche chez nos trois patients. La survenue de la gangrène était liée au garrot maintenu en place pendant au moins 24 heures. Dans la littérature le délai maximum pour l'ablation d'un garrot du membre supérieur est de 90 minutes, avec une tolérance en médecine pouvant atteindre 3 heures. Au-delà le patient est exposé à un risque d'ischémie puis de gangrène pouvant conduire à une amputation du membre [3]. Dans notre étude, l'erreur médicale a été en cause dans tous les trois cas comme dans l’étude de Doudou et al. [4] où un garrot oublié pendant 48 heures après un prélèvement sanguin au bras gauche a été responsable de la gangrène ischémique. Dans notre cas, l'oubli du garrot témoigne d'une négligence de la part de l'infirmier, d'une absence de surveillance du patient et de communication avec les parents, justifiant l'apparition de la gangrène ischémique. Les patients victimes étaient uniquement des nourrissons. kaushal et al [5] dans leurs études ont retrouvé que les patients d’âge pédiatrique étaient trois fois plus exposé aux erreurs médicales que les adultes. La vulnérabilité de cette tranche de patient serait liée à leur inaptitude à s'exprimer oralement. En Afrique au sud du Sahara surtout au Burkina les agents de santé de première ligne ignorent la déontologie et l’éthique médicale dans l'exercice de leurs fonctions. Ceci est d'autant entretenu du fait que les patients qui sont victimes d'erreurs ou de négligences médicales n'ont pas assez de connaissances sur leurs droits dans la relation médecin malade. Ces patients victimes de faute médicale ne peuvent pas définir s'il y'a eu faute ou pas et acceptent leur sort sans entreprendre de poursuites judiciaires pour réparation des préjudices. L'absence de sanction et l'insuffisance d'information sur les droits des patients et les obligations du personnel soignant aggravent ces dérives. En témoigne chez notre 1^er^ patient du fait de l'ignorance, les parents conscient de l'oubli du garrot n'ont pas attiré l'attention de l'agent de santé à temps. La faute médicale a été responsable d'une mutilation d'un membre thoracique chez deux patients, causant ainsi des préjudices fonctionnels, esthétiques et une restriction de leur avenir socioprofessionnelle. Les principes de sauvegarde de l'intérêt du patient qui devraient être connus de tous: principe de ne jamais nuire au malade, devoir d'assurer les soins nécessaires avec la plus grande conscience, mission d'assistance morale vis-à -vis du malade par une attitude correcte et compatissante [4] ont été bafoués. La prévention de telles fautes passent par outre la formation technique, la connaissance parfaite de la déontologie et de l’éthique médicale d'une part et d'autre part l’éducation et la sensibilisation de la population sur leurs droits en matière de santé [4, 6]. En outre l'amélioration de la sécurité des patients passe par la mise en place d'organismes chargés de la surveillance de la qualité des soins; l’établissement d'un climat de confiance entre soignant et soigné à travers une bonne stratégie de communication avec les patients sur leurs états de santé, les résultats de l'intervention médicale ainsi que les limites du traitement [6, 7]. La sanction des agents de santé pour faute médicale grave par les organismes de surveillance (ordre de médecin, ordre infirmier) et ou par la justice permettrait de resserrer l’étau chez les soignants en matière de qualité soins et de la sécurité du patient dans l'exercice de leur fonction.

## Conclusion

La gangrène ischémique iatrogène de membre par oubli de garrot en milieu hospitalier ne devrait pas se concevoir. Cela passe par la rigueur dans la formation technique, la sécurité dans l'administration des soins et une surveillance régulière et attentive des patients. Les sanctions des agents de santé par la justice et la réparation des dommages des patients victimes de faute médicale grave devraient être envisagées dans les pays en développement.
